# Leveraging MYC as a therapeutic treatment option for TNBC

**DOI:** 10.18632/oncoscience.424

**Published:** 2018-06-24

**Authors:** Jason P.W. Carey, Khandan Keyomarsi

**Affiliations:** Department of Experimental Radiation Oncology, The University of Texas MD Anderson Cancer Center, Houston, TX 77030, USA

**Keywords:** MYC, TNBC, PARP, biomarker, CDK

Women diagnosed with Triple Negative Breast Cancer (TNBC) have worse overall survival rates than other forms of breast cancer partly due to the absence of novel and targeted treatment modalities in this aggressive subtype [1]. The introduction of PARP inhibitors signifies a hope for individuals with germ-line BRCA mutations who typically present with TNBC, even though BRCA mutant patients represent a minor subset of the overall breast cancer population (∼10%) [2]. In the study “Synthetic lethality of PARP inhibitors in combination with MYC blockade is independent of BRCA status in triple negative breast cancer” we investigate the use of MYC as a treatment directed biomarker in TNBC that can predict outcome in response to standard of care and act as a target for the development of novel PARP inhibitor combination therapies [3].

As an influential oncogene, MYC can potentially regulate ∼over 4,000 genes at any given moment. Although the relationship between MYC and DNA repair has been investigated extensively, therapeutic modalities that exploit MYC directed DNA repair vulnerability are absent. Our study cultivates the direct link between MYC and DNA repair to exploit an increased response to PARP inhibitors [3]. Evaluation of TCGA datasets and an MD Anderson Pre/Post chemotherapy TNBC cohort validate the relationship between MYC and upregulation of the homologous recombination (HR) DNA repair signatures (e.g. Rad51) and correlate expression with decreased overall survival. Dual MYC/RAD51 expression dictated response to standard of care neo-adjuvant chemotherapy. MYC low/RAD51 low tumors demonstrated benefit to neo-adjuvant chemotherapy in regard to overall survival. Conversely, MYC High/RAD51high tumor demonstrated statistically significant worse overall survival in response to neo-adjuvant chemotherapy [3].

After reinforcing the relationship between MYC and the DNA repair pathway via RAD51, our study demonstrated a regulatory relationship between MYC and RAD51 gene expression [3]. *In vitro* and *in vivo* analysis of MYC downregulation via genomic and therapeutic inhibition induced PARP inhibitor sensitivity independent of BRCA status. The pan CDK (1, 2, 5, 9) inhibitor dinaciclib demonstrated superior efficacy versus other MYC inhibitors in combination with PARP inhibition. The use of MYC as a driver of biomarker targeted therapy for TNBC provides hope for a patient population lacking in therapeutic treatment options while also addressing a validated association between MYC and TNBC, with approximately ∼50% of TNBC patients demonstrating upregulation of MYC in tumors [3]. Our analysis along with other studies validates that MYC activation upregulates the HR DNA repair pathway. Conversely, the evaluation of de novo and acquired PARP inhibitor resistance mechanisms reinforce the reactivation of the HR pathway as an essential mediator of resistance [2].

**Figure 1:**
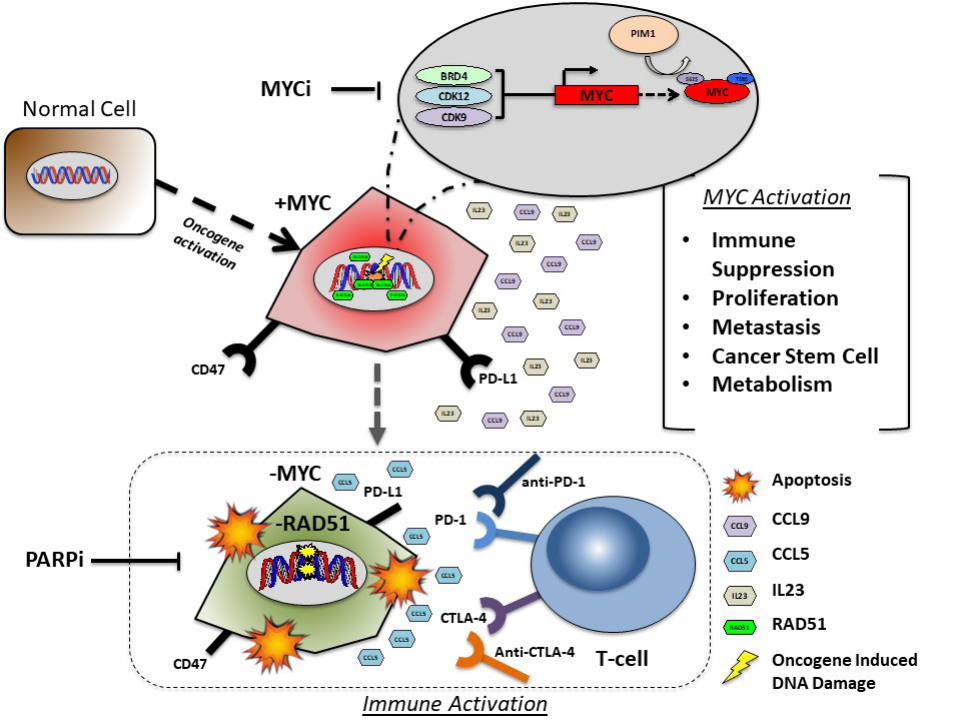
MYC activation regulates DNA repair and immune suppression in cancer cells Normal cell transformation via MYC oncogene activation initiates replication stress induced DNA damage that is subsequently repaired by MYC regulated RAD51 dependent homologous recombination DNA repair. MYC activates several hallmarks of cancer including increased proliferation, metastasis, the cancer stem cell (CSC) phenotype and altered metabolism. MYC elicits immune suppression via CD47 and PD-L1 upregulation with subsequent upregulation of CCL9 and CCL5 cytokines. Pharmacological inhibition of MYC regulators (BRD4, CDK12, CDK9, PIM1) down regulates MYC expression in cancer cells with subsequent downregulation of HR DNA repair and increased sensitivity to PARP inhibition induced cell death. MYC inhibition activates CCL5 secretion, accompanied by immune recruitment and activation in the tumor microenvironment.

The relationship between CDK inhibition and MYC expression has illuminated a regulatory role of the cell cycle over MYC expression (Figure 1). Additionally, CDK12 inhibition induces PARP inhibitor sensitivity via downregulation of HR [4]. CDK12 inhibition may induce PARP inhibitor sensitivity via MYC regulation. DNA Repair protein RAD51 can augment response of PARP inhibitors in BRCA mutant cell lines by rescuing HR defects and promoting PARP inhibitor resistance [2]. Additionally ectopic RAD51 expression fosters increased metastasis/EMT in TNBC cell lines, via upregulation of the cancer stem cell phenotype a trait that correlates with increased MYC expression [2].

Our study also alludes to the evolution of PARP inhibitors in the clinical setting beyond BRCA status. Respected for their efficacious treatment responses in HR defect tumors with measurably decreased adverse events versus standard chemotherapy, PARP inhibitors offer a new horizon for cancer treatment [2]. The shift to broaden the scope of PARP inhibitors beyond both BRCA mutant and HRD defects has begun in ovarian cancer with 3 PARP inhibitors being FDA approved for single agent activity in platinum sensitive patients [2].

More recently, Sun et al. demonstrated that BRD4 inhibition induces PARP inhibitor sensitivity in ovarian cancers independent of BRCA status [5]. Our study validated bromodomain inhibitor JQ1 and PARP inhibition as a synergistic combination therapy in TNBC. Although this study suggests the CtIP (C-terminal binding protein interacting protein) acts as the lynchpin in dual BRD4-PARP inhibition, MYC may serve as a biomarker of drug activity. The investigation into novel targeted therapies to combine with PARP inhibitors has highlighted HSP90, EGFR and WEE1 Kinase as additional synergistic combinations that increase PARP inhibitor efficacy [2].

To further advance the clinical application of PARP inhibitors, the investigations into novel immune-oncology agents are promising (Figure 1). Studies have focused on PARP inhibition in combination with several checkpoint inhibitors targeting PD-1, PD-L1 and CTLA-4-1 [6]. Moreover, our study demonstrates that dinaciclib downregulated PD-L1 *in vivo*. The down regulation of PD-L1 signals that checkpoint inhibitors may work in combination with dual dinaciclib + PARP inhibition. Two recent studies support the role that MYC plays in immune regulation; specifically MYC cooperates with KRAS to drive tumor growth via IL-23 and CCL9 immune suppression [7]. In another study by Topper et al dual DMNT & HDAC inhibition downregulates MYC, while activating the immune system via IFNγ signaling resulting in the upregulation of immune activation chemokine CCL5 [8]. These studies point to a hierarchal role of MYC in regulating immune suppression in cancer cells thus providing the opportunity for novel combinations (e.g. anti PD-1 or CTLA-4) that exploit MYC inhibition while promoting immune activation.

Dinaciclib is a pan-CDK inhibitor that has not made it past Phase 2 clinical trial evaluation due to high toxicity [3]. The reality remains that dinaciclib will probably remain a non-viable therapeutic option for patients moving forward due to lack of efficacy as a single agent and a challenged clinical profile [3]. However, the list of therapeutic agents that downregulate MYC in various cancer models is rapidly expanding. Combination therapeutic approaches promise a viable option moving forward regarding MYC inhibition in combination with PARP inhibitors.

As we search for novel combination therapies that increase the efficacy and scope of the patient population eligible for PARP inhibitor treatment, it is paramount to remain focused on a clearly defined outcome. The use of efficacious biomarkers such as MYC that dictate response to therapy is essential to the evolution of targeted treatment option for TNBC patients (Figure 1). We understand the significance of PARP inhibitors as a scientific discovery and now the challenge lies within out ability improve upon the science.
